# The equity impact of community women’s groups to reduce neonatal mortality: a meta-analysis of four cluster randomized trials

**DOI:** 10.1093/ije/dyx160

**Published:** 2017-08-25

**Authors:** Tanja A J Houweling, Caspar W N Looman, Kishwar Azad, Sushmita Das, Carina King, Abdul Kuddus, Sonia Lewycka, Dharma S Manandhar, Neena Sah More, Joanna Morrison, Tambosi Phiri, Shibanand Rath, Mikey Rosato, Aman Sen, Prasanta Tripathy, Audrey Prost, David Osrin, Anthony Costello

**Affiliations:** 1 Department of Public Health, Erasmus MC University Medical Center Rotterdam, Rotterdam, The Netherlands; 2 Institute for Global Health, University College London, London, UK; 3 Perinatal Care Project (PCP), Diabetic Association of Bangladesh (BADAS), Dhaka, Bangladesh; 4 Mother and Infant Research Activities (MIRA), YB Bhawan, Kathmandu, Nepal; 5 Ekjut, Potka, Chakradharpur, Jharkhand, India; 6 MaiMwana Project, Mchinji, Malawi; 7 Society for Nutrition, Education and Health Action (SNEHA), Shahunagar, Mumbai, India; 8 Nuffield Department of Medicine, Centre for Tropical Medicine, University of Oxford, Oxford, UK

**Keywords:** Developing countries, obstetric, infant mortality, intervention studies, inequalities

## Abstract

**Background:**

Socioeconomic inequalities in neonatal mortality are substantial in many developing countries. Little is known about how to address this problem. Trials in Asia and Africa have shown strong impacts on neonatal mortality of a participatory learning and action intervention with women’s groups. Whether this intervention also reduces mortality inequalities remains unknown. We describe the equity impact of this women’s groups intervention on the neonatal mortality rate (NMR) across socioeconomic strata.

**Methods:**

We conducted a meta-analysis of all four participatory women’s group interventions that were shown to be highly effective in cluster randomized trials in India, Nepal, Bangladesh and Malawi. We estimated intervention effects on NMR and health behaviours for lower and higher socioeconomic strata using random effects logistic regression analysis. Differences in effect between strata were tested.

**Results:**

Analysis of 69120 live births and 2505 neonatal deaths shows that the intervention strongly reduced the NMR in lower (50–63% reduction depending on the measure of socioeconomic position used) and higher (35–44%) socioeconomic strata. The intervention did not show evidence of ‘elite-capture’: among the most marginalized populations, the NMR in intervention areas was 63% lower [95% confidence interval (CI) 48–74%] than in control areas, compared with 35% (95% CI: 15–50%) lower among the less marginalized in the last trial year (*P*-value for difference between most/less marginalized: 0.009). The intervention strongly improved home care practices, with no systematic socioeconomic differences in effect.

**Conclusions:**

Participatory women’s groups with high population coverage benefit the survival chances of newborns from all socioeconomic strata, and perhaps especially those born into the most deprived households.

**Key Messages**

- This is one of the first studies to report on the equity impact of an intervention on neonatal mortality across socioeconomic strata.
- Based on a meta-analysis of four cluster randomized controlled trials, including 69120 live births and 2505 neonatal deaths, we find that community-based women’s groups reduced neonatal mortality equitably and substantially across socioeconomic strata. This contrasts with concerns about elite capture of community-based interventions and with evidence that health interventions generally benefit the better-off most.
- We conclude that community-based women’s groups can substantially contribute to the survival of all newborns, perhaps especially of disadvantaged infants, and to progress towards the targets set in the Every Newborn Action Plan.

## Introduction

A world without preventable newborn deaths, as envisaged in the Every Newborn Action Plan led by WHO and UNICEF, is only achievable when strategies are available to close the gap in newborn mortality between socioeconomic groups.^1^^,^^2^ The odds of surviving the first month of life are grossly unequal between infants born in poor and rich households and to higher and less educated mothers within the same country.^3–5^ Unfortunately, little is known about what works to close these mortality gaps.

The strongest evidence on intervention effects comes from randomized controlled trials (RCT). Four cluster randomized controlled trials, in Asia and Africa, have shown that community-based women’s groups can substantially reduce neonatal mortality, perhaps especially through improved home care practices, provided that coverage of the women’s groups in the population is sufficiently high (≥ 30% of pregnant women participating in groups).^6^ These trials provide an opportunity to assess the equity impact of community-based interventions on neonatal mortality using a gold standard study design. Whereas community-based interventions run the risk of elite capture, in which the best-off reap more benefits,^7–11^ there are indications that community-based women’s groups can reduce mortality inequalities by reaching all socioeconomic strata through their soft-targeting approach (i.e. tailoring the intervention design to the target group rather than applying strict in-/exclusion criteria).^12^^,^^13^ The equity impact of the groups has, however, not yet been evaluated systematically across the trials, and a meta-analysis will generate evidence on approaches that may reduce inequalities in neonatal mortality.

In this study, we analysed the effect of community-based women’s groups on neonatal mortality, health care use and home care practices in lower and higher socioeconomic strata, and tested for differences in effect between strata, using data from four RCTs.

## Methods

We included all four RCTs of community-based women’s groups that had sufficient population coverage to be able to have an effect on the neonatal mortality rate (NMR). These studies were identified in two previously published systematic reviews and a meta-analysis.^6^^,^^14^ That meta-analysis, and a corresponding heterogeneity test, have shown that the women’s group intervention only has an effect on neonatal mortality when coverage is high. Three women’s group trials had had too low coverage to be able to have an effect on NMR. In those trials, 1%, 3% and 10% of pregnant women in intervention areas attended groups, respectively,^15–17^ compared with around 35–50% in the other trials.^6^ Given that we were interested in the distribution of the substantial mortality effects shown in the highly effective women’s groups interventions, we considered further sub-group analyses of the low-coverage trials a priori as futile.

Nonetheless, we conducted a secondary analysis of all six published trials that contained data on socioeconomic position, i.e. including the four ‘high coverage trials’^18–21^ and two ‘low coverage trials’.^15^^,^^16^ In a third ‘low coverage trial’,^17^ no information on socioeconomic position was collected, so the trial had to be excluded. We also had to exclude a trial in Dhanusha district in Nepal, of which the findings have not yet been published.^22^

The four ‘high coverage trials’ were located in rural areas of Nepal, India, Bangladesh and Malawi. One ‘low coverage trial’ was located in rural Bangladesh, and one in urban India (Mumbai). In the trial sites, geographical clusters were randomly allocated to the intervention or control arm (Table S1, available as Supplementary data at *IJE* online). In the intervention areas, participatory learning and action groups were set up. The groups identified and prioritized maternal and newborn health problems, and developed and evaluated strategies to address them. Strategies included, among others, home visits to pregnant women, emergency funds, arrangements for emergency transport to a health facility, and the preparation and distribution of safe delivery kits. The groups met monthly under the guidance of a facilitator for 2–3 years. The intervention and evaluation design were similar across the study sites.^6^ This intervention led to a reduction in neonatal mortality of around 30–40% when intervention coverage was high.^6^^,^^18–21^

The women’s groups are a community-based intervention, in which active engagement of the wider community played an important role. The women’s groups, for example, organized community meetings in which they explicitly asked support from the wider community for the implementation of their strategies. The strategies were meant to benefit all pregnant women, also those not attending groups. Women’s group members, for example, made home visits to women who were not members, to tell them about what they have learned in the group and/or to support them during delivery and the newborn period. Therefore, intervention effects are expected to be seen community-wide, not just among women’s group members.

The trials were conducted in large surveillance sites where the total population was prospectively followed up and all births and birth outcomes were registered. Identification was done by local key informants who typically covered 200–250 households. Interviewers verified the key informants’ information and interviewed all mothers around 6 weeks postpartum.^23^^,^^24^ We obtained the full individual-level trial datasets from the trial analyst or database manager and included all live births to residents of the study areas. For the Bangladesh trial, the migrant tea garden populations, which were socioculturally distinct from the other study areas, were excluded, following the original trial paper (we examined sensitivity of our main findings against the in/exclusion of the tea garden population and found that findings remained very similar).

We examined intervention effects on NMR (number of deaths in the first 28 days of life/1000 live births) and health care use and home care practices (i.e. the primary and secondary outcomes of the original trials) among lower and higher socioeconomic strata. The strongest effects were generally reported in the final trial year, probably due to a lag time during which the groups become established and active. Given our interest in the distribution of these strong effects, we conducted our main analysis for the final year, as a priori planned, and secondary analyses for the last two trial years (time window for evaluation in several trials).

The equity impact was examined for dimensions of socioeconomic position that were relevant across all sites: literacy (can read vs cannot read, based on respondent’s ability to read a brief passage) and economic status. Household ownership of assets, combined into an index using principal component analysis for each trial, was used as indicator of economic status. A high percentage of births (50–60% in the Nepal and India trials) took place in households with none or few of the asset items for which data were available. Based on their asset index score, live births were categorized into two roughly equally sized groups, poor and less poor, using Stata’s quantile function. For the Nepal trial, we were restricted by the pre-defined asset levels in the postpartum interview, which we combined into two wealth groups (poorest: owns none of the asset items; less poor: owns at least one asset item) (see Table 1). Given our interest in intervention effects on the bottom end of the socioeconomic ladder, we also constructed a multidimensional measure of socioeconomic position, including both literacy and economic status, with those who were illiterate and poor categorized as ‘most marginalized’ and the rest of the population as ‘less marginalized’. The definitions of these dimensions of socioeconomic position were specified in advance of the analysis.

**Table 1.** Number of live births and neonatal deaths in intervention and control areas, baseline and trial periods combined

	Nepal^a^						India						Bangladesh						Malawi						Total	
	Control			Intervention			Control			Intervention			Control			Intervention			Control			Intervention				
	Live births	%	Neonatal deaths	Live births	%	Neonatal deaths	Live births	%	Neonatal deaths	Live births	%	Neonatal deaths	Live births	%	Neonatal deaths	Live births	%	Neonatal deaths	Live births	%	Neonatal deaths	Live births	%	Neonatal deaths	Live births	Neonatal deaths
Total	3264	100	121	2923	100	78	10981	100	634	11735	100	542	13373	100	411	13647	100	359	6776	100	202	6421	100	158	69120	2505
Marginalization																										
Less marginalized	1727	55	56	1846	65	45	5675	52	297	4668	40	205	11084	83	330	10986	81	269	5162	77	152	4964	78	127	46112	1481
Most marginalized	1433	45	60	996	35	32	5296	48	337	7063	60	337	2283	17	81	2655	19	89	1562	23	50	1400	22	31	22688	1017
Literacy																										
Literate	717	23	25	1111	40	19	3363	31	170	2795	24	112	10237	77	289	10199	75	249	4123	61	116	4101	65	112	36646	1092
Illiterate	2364	77	91	1668	60	56	7598	69	462	8932	76	429	3133	23	122	3437	25	109	2590	39	86	2257	35	46	31979	1401
Economic status																										
Less poor	1437	44	46	1427	49	36	4570	42	232	3391	29	142	6386	48	171	5959	44	128	3021	45	86	2654	42	51	28845	892
Poorest	1818	56	75	1484	51	42	6411	58	402	8343	71	400	6978	52	240	7684	56	230	3692	55	115	3700	58	106	40110	1610

Summing of the sub-groups does not always add up to the totals because of missing values.

Note on measurement of economic status: Nepal: predefined asset levels in the surveillance questionnaire, based on household ownership of one or more of the items in the list, were as follows: richest (bus, truck, motorcycle, TV, motor tractor, fridge, hand tractor), next-rich (sewing machine, cassette player, fan, radio, camera, bicycle), next-poor (wall clock, iron), poorest (none of the above). As over 50% of live births were in households that owned none of the asset items, we combined the richest, next-rich and next-poor level into the category ‘less poor’. India, Bangladesh, Malawi: principal component analysis-based asset index. List of asset items for which data were available varied between trials: India (electricity, generator, battery, fan, TV, radio, tape, fridge, bicycle, motor), Bangladesh (electricity, generator, fan, TV, radio, fridge, bicycle, telephone), Malawi (electricity, radio, bicycle, motor, car, lamp, oxcart).

^a^For the original Nepal trial paper, data entry was frozen on 1 December 2003. However, after this date, the monitoring and evaluation team continued to receive a few additional forms for births that took place within the trial period (38 live births, of which 2 NND in control areas, and 24 live births of which 2 NND in intervention areas). We included the data for these records in our analysis. Although the inclusion of these births that took place within the trial period is certainly more accurate, the very small difference in numbers of records will have had no effect on the findings.

### Statistical analyses

In sum, we obtained our findings using logistic regression analysis in which we adjusted for the trials’ sampling schemes. The clustered design of the trials was adjusted for using random effects modelling; for trials with a stratified design (India, Bangladesh), we included stratum as covariate. We checked the quadrature approximation used in the random effects estimators using the quadchk command in Stata.

For the Nepal trial, with pair-matched clusters, we included a fixed effect for matching pair in the models. In the Malawi trial, which used a factorial design, the mortality effect of the intervention was based on a ‘treatment group analysis’, comparing the ‘women’s group only’ arm with the ‘no intervention’ arm. We replicated this in our analysis.

The original trial papers usually estimated the difference in log odds of neonatal death between intervention and control arm at endline only, adjusted for sampling design, essentially using the following model:

(1) estimated log odds NND=α+β1Interv.Arm+β2Strata +ui NND∼Bernoulli ui∼N

Interv.Arm is the dummy for intervention vs control arm. β_1_ represents the difference in log odds of neonatal death (NND) between intervention and control arm at endline, β_2_ the adjustment for the stratified sampling design and u the random effect term for cluster.

We expanded this by adding information about baseline neonatal mortality, thereby adjusting for the difference in odds of neonatal death between intervention and control arm at baseline (equation 2):

(2) estimated log odds NND=α+β1Interv.Arm+β2Endline +β3Interv.Arm*Endline+β4Strata+ui NND∼Bernoulli ui∼N

‘Endline’ is the dummy for endline vs baseline. β_1_ now stands for the difference in log odds of NND between intervention and control arm at baseline, β_2_ for the difference in log odds of NND between endline and baseline in the control arm, and β_3_ for the effect of the intervention, i.e. difference in log odds of NND between intervention and control arm at endline, adjusted for baseline differences in neonatal mortality. The exponent of β_3_ provides the odds ratio (OR) for the intervention effect in the total population adjusted for baseline differences.

As most studies had more than one intervention year, we expanded equation 2 by distinguishing the intervention effect between follow-up years (equation 3):

(3) estimated log odds NND=α+β1Interv.Arm+β2yYear +β3yInterv.Arm*Year+β4Strata+ui NND∼Bernoulli ui∼N

Year is the categorical variable for the separate intervention years, where β_2y_ splits up β_2_ into two or three (depending on the number of intervention years) separate effects of year. β_3y_ represents the difference in log odds of NND between intervention and control arm in year y, adjusted for baseline differences. For example, β_33_ gives the difference in log odds of NND between intervention and control arm in year 3 (adjusted for baseline differences). The exponents of β_3y_ are given in the first row of Table 3 and provide the intervention effects for the total population, adjusted for baseline mortality differences between intervention and control.

Then, we estimated the intervention effects for each socioeconomic stratum, while adjusting for baseline differences between intervention and control arm in socioeconomic inequality in neonatal mortality (equation 4):

(4) estimated log odds NND=α+β1Interv.Arm+β2yYear +β3yInterv.Arm*Year+β4SEP_L+β5SEP_L*Year+β6SEP_L*Interv.Arm +β7ySEP_L*Interv.Arm*Year+β8Strata+ui NND∼Bernoulli ui∼N

**Figure 1:**
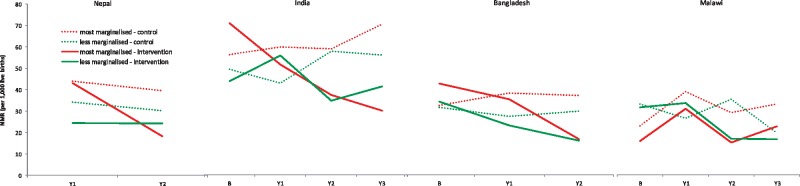
Trends in the neonatal mortality rate (NMR) in intervention and control areas for the most and less marginalised, four women’s group trials. *Note:* women who were illiterate and poor were categorised as ‘most marginalised’; the rest of the population was categorised as ‘less marginalised’. B: baseline. Y1: intervention year 1, Y2: intervention year 2, Y3: intervention year 3.

SEP_L is the dummy for low vs high socioeconomic position. Now, β_1_ represents the difference in log odds of NND between intervention and control arm at baseline for the high socioeconomic group (the reference group); β_2y_ the effect of the different years in the control arm for the high socioeconomic group; β_3y_ the difference in odds of NND between intervention and control arm in year y for the high socioeconomic group, adjusted for baseline differences in log odds of NND (i.e. the intervention effect in year y for the high socioeconomic group adjusted for mortality baseline differences); β_4_ the difference in log odds of NND between low and high socioeconomic position at baseline; β_5_ the difference in effect of year between low and high socioeconomic position in the control arm; β_6_ the difference between low and high socioeconomic position in log odds of NND between intervention and control arm at baseline; and β_7_ the difference in intervention effect in year y between low and high socioeconomic position (adjusted for baseline mortality differences). The *P*-values for β_7_ are those for the difference in intervention effect between low and high socioeconomic position as given in Table 3. The exponent of β_3_ represents the intervention effect in the high socioeconomic group and the exponent of (β_3__ __+__ _β_7_) represents the intervention effect in the low socioeconomic group as given in Table 3. We transformed the estimated log odds into NMR by socioeconomic group by year and trial arm, as given in Table 2 and Figure 1.

**Table 2.** Neonatal mortality rate (per 1000 live births) for baseline and intervention years, for intervention and control areas, four women’s group trials

	Nepal				India								Bangladesh						Malawi							
	Control		Intervention		Control				Intervention				Control			Intervention			Control				Intervention			
	Y1	Y2	Y1	Y2	B	Y1	Y2	Y3	B	Y1	Y2	Y3	B	Y1	Y2	B	Y1	Y2	B	Y1	Y2	Y3	B	Y1	Y2	Y3
Total	40	33	31	21	52	51	58	63	60	53	36	35	32	29	31	36	26	16	30	29	34	22	28	33	16	18
Marginalization																										
Less marginalized	34	30	25	24	49	43	58	56	44	56	35	41	32	27	30	34	23	16	33	26	35	20	31	33	17	17
Most marginalized	44	39	43	18	56	60	59	70	71	52	37	30	32	38	37	43	35	17	23	39	29	33	16	31	15	23
Literacy																										
Literate	38	32	18	16	44	42	54	59	36	51	37	34	29	26	29	34	23	16	31	25	34	18	35	36	17	17
Illiterate	40	36	40	26	55	55	60	64	67	54	36	35	39	41	36	42	33	16	30	36	33	29	16	27	15	19
Economic status																										
Less poor	33	30	24	27	49	44	56	51	49	57	26	40	27	25	28	33	21	11	35	27	28	20	18	25	17	15
Poorest	46	36	38	16	55	56	60	72	65	52	40	33	36	33	33	38	29	20	27	31	38	24	35	39	16	20

B, baseline; Y1, intervention year 1; Y2, intervention year 2; Y3, intervention year 3.

**Table 3.** Intervention effects on the neonatal mortality rate for lower and higher socioeconomic groups, per trial and pooled estimates, for the last study year

	Pooled estimates			Nepal			India			Bangladesh			Malawi		
	OR^a^	95% CI	*P*- value^b^	OR^a^	95% CI	*P*- value^b^	OR^a^	95% CI	*P*- value^b^	OR^a^	95% CI	*P*- value^b^	OR^a^	95% CI	*P*- value^b^
Total	0.51	(0.42–0.63)	0.000	0.64	(0.40–1.03)	0.064	0.46	(0.32–0.65)	0.000	0.45	(0.31–0.64)	0.000	0.80	(0.43–1.51)	0.498
Marginalization															
Less marginalized	0.65	(0.50–0.85)	0.009	0.82	(0.45–1.50)	0.240	0.82	(0.48–1.41)	0.010	0.49	(0.32–0.73)	0.413	0.85	(0.43–1.70)	0.652
Most marginalized	0.37	(0.26–0.52)		0.45	(0.20–1.01)		0.32	(0.20–0.51)		0.33	(0.14–0.76)		0.67	(0.25–1.79)	
Literacy															
Literate	0.56	(0.41–0.76)	0.587	0.50	(0.20–1.27)	0.551	0.71	(0.33–1.50)	0.240	0.46	(0.30–0.70)	0.798	0.91	(0.43–1.94)	0.547
Illiterate	0.50	(0.37–0.66)		0.70	(0.40–1.23)		0.42	(0.28–0.63)		0.41	(0.20–0.84)		0.66	(0.27–1.59)	
Economic status															
Less poor	0.58	(0.42–0.80)	0.360	0.91	(0.47–1.75)	0.143	0.76	(0.41–1.42)	0.055	0.31	(0.17–0.57)	0.120	0.75	(0.30–1.88)	0.864
Poorest	0.48	(0.36–0.62)		0.44	(0.22–0.90)		0.36	(0.24–0.56)		0.57	(0.36–0.90)		0.83	(0.39–1.76)	

^a^The ratio of the odds of neonatal mortality in the intervention compared with the control areas adjusted for baseline differences. For the Nepal trial, it was not possible to adjust for baseline mortality differences because of the absence of prospectively collected baseline data.

^b^*P*-value for the test on difference in OR between lowest and highest socioeconomic groups. For the total population, it gives the *P*-value for the difference between intervention and control. *P*-values for heterogeneity test in pooled analysis: marginalization (*P* = 0.3212), literacy (*P* = 0.3548), economic status (*P* = 0.04148). Absolute intervention effects for last study year (per 1000 live births): India, less marginalized −9 (95% CI: −32;15), most marginalized: −55 (95% CI: −78;−30), *P*-value for difference: 0.012. Nepal, less marginalized −5 (95% CI: −21;11), most marginalized −21 (95% CI: −42;0), *P*-value for difference: 0.212. Bangladesh, less marginalized −17 (95% CI: −27;−6), most marginalized −31 (95% CI: −53;−7), *P*-value for difference: 0.272. Malawi, less marginalized −3 (95% CI: −11;6), most marginalized −10 (95% CI: −29;11), *P*-value for difference: 0.542.

For the Nepal trial, formal correction for baseline differences in NMR was impossible because there were no prospective baseline data. The retrospective baseline assessment (i.e. based on birth history data) in this trial showed no NMR differences between arms but lower rates than subsequently measured using prospective surveillance. The ORs for Nepal in Table 3 therefore give the ratio of the odds of neonatal death in intervention areas compared with control areas in the last study year, only adjusting for clustering and the pair-matched design. For Malawi (and urban India, Table S2, available as Supplementary data at *IJE* online) we adjusted for cluster-level average mortality rates at baseline, analogous to the original trial paper.

Using the same models as above, we also examined the intervention effects on health care use and home care practices in lower and higher socioeconomic groups. We used Stata 12 for the above analyses (Stata, College Station, TX, USA).

We also estimated the absolute intervention effects (per 1000 live births) for low and high SEP. We used a generalized linear mixed model to estimate mortality probabilities in a hypothetical new random cluster, for each of the strata or matched pairs. We combined the mortality predictions into average mortality probabilities weighted by the number at risk in each of the strata or matched pairs. Subtracting these averaged probabilities([intervention minus control at endline] minus [intervention minus control at baseline]) then provides the absolute intervention effect. A non-parametric bootstrap was done to get confidence intervals around the absolute effects (1000 replicas).

After we conducted the analyses for each trial separately, we used the beta and standard error of the effect estimate per trial to estimate pooled effects for all trials combined. We used random effects models for the meta-analysis, with trial as random effect, because we assumed that the (differential) effects of each trial were taken from an underlying distribution. The pooled analyses were replicated for early and late NMR. These analyses were conducted in R 3.0.1 (library lme4).

Approval for the trials was received from the Research Ethics Committee at the UCL Institute of Child Health and from appropriate national ethics committees.

## Results

There were 69120 live births and 2505 neonatal deaths in the trial areas during the trial periods (Table 1). Births in Nepal and India were largely to illiterate women (60–77%), in contrast to Bangladesh and Malawi (23–39%). Of the study populations, 50–70% was categorized as poor. The percentage categorized as most marginalized varied from around 20% (Bangladesh, Malawi) to around 40% in Nepal and 55% in India. Despite the random allocation of clusters to the intervention and control arm, the population in the intervention arm in India was somewhat worse off compared with the control arm; in Nepal this was the other way around.

Baseline NMR varied from 30–40/1000 (Malawi, Bangladesh, Nepal) to 50–60/1000 (India) (Table 2, Figure 1). In India and Nepal, NMR declined more among lower socioeconomic strata than among higher strata in intervention areas. In control areas, NMR declined more slowly, especially among lower strata (Nepal), or even increased (India). In Bangladesh, NMR declined strongly in all strata in intervention areas, and remained roughly stable in control areas. In Malawi, NMR declined in intervention areas in years 2–3 in lower and higher socioeconomic groups and in control areas in year 3 in higher strata.

The meta-analysis shows that the intervention reduced NMR substantially and equitably across lower and higher socioeconomic groups (Table 3). Intervention effects ranged from a 35–44% mortality reduction in higher strata to a 50–63% reduction in lower strata in the last study year, depending on the measure of socioeconomic position used [last 2 years: 32–39% and 40–48%, respectively (Table S3, available as Supplementary data at *IJE* online)]. The effects in lower strata were as strong as or stronger than those in higher strata. In the last year, the effect was much stronger among the most marginalized (63% mortality reduction, OR 0.37; 95% CI: 0.26–0.52) than among the less marginalized (35% mortality reduction, OR 0.65; 95% CI: 0.50–0.85) (*P*-value for difference: 0.009), whereas the effects were similar by literacy and economic status. In the last 2 study years, effects were similar in lower and higher strata irrespective of the measure of socioeconomic position (SEP) used. In the individual trials, there was a tendency for stronger effects among lower socioeconomic groups, especially in the India trial, whereas the effects in the other trials were comparable across strata and/or confidence intervals were too large to detect differential effects. There was no heterogeneity between trials in the differential effect of the intervention on lower and higher socioeconomic groups, except when stratifying by economic status. The meta-analysis for all six trials, including the two trials with very low women’s group coverage and no intervention effect, was comparable to the results for the four trials presented above, albeit with point estimate closer to one [i.e. less marginalization OR (95% CI): 0.84 (0.64–1.10); more marginalized OR (95% CI): 0.48 (0.30–0.79), *P* = 0.056, see Table S2).

When examining absolute intervention effects (per 1000 live births), again there was a tendency for stronger effects among the most marginalized compared with the better-off in all trials; but only for the India trial, the *P*-value for the difference in effect between strata was small (*P* = 0.012, Table 3 footnote). The intervention in India was associated with an NMR change of −55/1000 (95% CI: −78 to −30) among the most marginalized and −9/1000 (95% CI: −32 to 15) in the less marginalized.

For early NMR, substantial effects were observed among lower and higher socioeconomic strata in the pooled analysis, with mortality declines of around 50% to 61% among lower strata and 44% to 53% among higher strata depending on the measure of socioeconomic position used (Table 4). No differential effects between lower and higher strata were detected. For late NMR, strong effects were only detected for lower strata (poorest: 56% mortality decline (95% CI: 29 73%), illiterate: 57% (95% CI: 29–74%), most marginalized: 72% (95% CI: 48–85%)), whereas the ORs for late NMR among higher strata were close to one. The *P*-values for the differences in effect on late NMR between lower and higher strata were mostly small (0.003 for marginalization, 0.051 for economic status, 0.107 for literacy). In the last 2 study years, the effects on early NMR were comparable across socioeconomic strata, but there was a tendency for stronger effects on late NMR among lower strata (results not shown).

**Table 4.** Pooled effect estimates for the early and late neonatal mortality rate (NMR) for lower and higher socioeconomic groups, for the last study year

	Pooled estimates, early NMR			Pooled estimates, late NMR		
	OR^a^	95% CI	*P*-value^b^	OR^a^	95% CI	*P*-value^b^
Total	0.47	(0.37–0.61)	0.000	0.58	(0.40–0.84)	0.004
Marginalization						
Less marginalized	0.56	(0.41–0.77)	0.171	0.93	(0.58–1.49)	0.003
Most marginalized	0.39	(0.26–0.59)		0.28	(0.15–0.52)	
Literacy						
Literate	0.47	(0.32–0.68)	0.783	0.80	(0.45–1.41)	0.107
Illiterate	0.50	(0.36–0.71)		0.43	(0.26–0.71)	
Economic status						
Less poor	0.47	(0.31–0.70)	0.929	0.94	(0.52–1.69)	0.051
Poorest	0.48	(0.35–0.66)		0.44	(0.27–0.71)	

^a^The ratio of the odds of neonatal mortality in the intervention compared with the control areas adjusted for baseline differences.

^b^*P*-value for the test on difference in OR between lowest and highest socioeconomic groups. For the total population, it gives the *P*-value for the difference between intervention and control.

Among infants of women in the intervention arm who had not attended the groups, the (differential) effects on neonatal mortality were similar to, or only slightly smaller than, those in the full trial populations (Table S4, available as Supplementary data at *IJE* online).

Only the Nepal trial showed strong effects on antenatal and delivery care, in all strata (Table 5). The Bangladesh trial showed some improvements for the whole population and higher strata in health care use during the antenatal period [antenatal care (ANC) 3+, medical treatment for ill pregnant women], but not during delivery. No effects on health care use were observed in the trials in India and Malawi. The patterns for the last 2 trial years were similar to those in the last year (results not shown).

Conversely, effects on home care practices were substantial in all three Asian trials, especially for hygienic practices around delivery and for delayed bathing. The India and Bangladesh trials also showed strong effects on thermal care and breastfeeding. These effects were observed in lower and higher strata, with no systematic differences in effect. No effects on home care practices were observed in Malawi.

**Table 5.** Intervention effects on behaviour for lower and higher socioeconomic groups per trial, for the last study year

	Nepal							India						
	Total		High SEP		Low SEP		Difference	Total		High SEP		Low SEP		Difference
	OR*	95% CI	OR*	95% CI	OR*	95% CI	*P*-value*	OR*	95% CI	OR*	95% CI	OR*	95% CI	*P*-value*
Health care use														
3 + antenatal care visits to medical provider	4.10	(2.07–8.14)	4.18	(2.09–8.36)	3.19	(1.50–6.79)	0.2510	1.12	(0.92–1.35)	1.02	(0.78–1.32)	1.10	(0.81–1.48)	0.715
Medical treatment sought for illness during pregnancy	4.79	(2.85–8.05)	6.90	(3.55–13.38)	2.35	(1.13–4.90)	0.0160	0.79	(0.64–0.98)	0.76	(0.57–1.02)	0.73	(0.53–1.02)	0.893
Institutional delivery	5.88	(2.57–13.46)	6.24	(2.60–14.99)	4.94	(1.43–17.06)	0.7300	0.87	(0.70–1.10)	0.79	(0.59–1.06)	1.03	(0.69–1.52)	0.299
Institutional delivery for prolonged labour	8.69	(2.25–33.61)	10.50	(2.54–43.29)	6.02	(1.14–31.78)	0.4930	1.02	(0.53–1.97)	1.51	(0.60–3.79)	0.61	(0.23–1.62)	0.176
Home care: antenatal practices														
Iron intake	0.46	(0.23–0.91)	0.43	(0.21–0.91)	0.55	(0.22–1.33)	0.6150	1.47	(1.23–1.76)	1.24	(0.94–1.64)	1.63	(1.29–2.06)	0.141
Home care: delivery practices														
Birth attendant washed hands	6.95	(3.16–15.29)	6.71	(3.02–14.95)	7.22	(3.14–16.57)	0.7790	4.19	(3.38–5.21)	4.73	(3.43–6.53)	3.85	(2.86–5.18)	0.358
Clean delivery kit used	5.90	(3.72–9.36)	5.01	(3.10–8.10)	7.74	(3.58–16.73)	0.2750	3.81	(2.89–5.02)	5.53	(3.68–8.31)	2.18	(1.44–3.30)	0.002
Plastic sheet used	–							3.40	(2.38–4.85)	5.65	(3.37–9.45)	1.92	(1.12–3.28)	0.004
Cord cut with new or boiled blade	3.06	(1.78–5.26)	3.36	(1.93–5.85)	2.55	(1.38–4.72)	0.2740	2.15	(1.72–2.69)	2.59	(1.74–3.85)	1.96	(1.48–2.58)	0.258
Cord tied with boiled thread	–							7.90	(5.92–10.53)	9.46	(6.29–14.23)	5.84	(3.80–8.97)	0.110
Home care: postnatal practices														
Appropriate cord care (nothing applied on stump)	1.28	(0.53–3.08)	1.07	(0.44–2.64)	1.53	(0.61–3.86)	0.1290	1.86	(1.41–2.47)	1.34	(0.88–2.02)	2.42	(1.66–3.53)	0.037
Baby wrapped or put on the skin within 10 min								7.58	(5.20–11.05)	4.10	(2.37–7.11)	11.74	(7.03–19.60)	0.006
Baby placed on mother's skin within 30 min	0.47	(0.17–1.32)	0.44	(0.16–1.23)	0.37	(0.11–1.20)	0.6880	0.96	(0.71–1.30)	0.63	(0.39–1.02)	1.26	(0.85–1.87)	0.030
Baby not bathed in first 6 h after birth	3.95	(2.42–6.46)	3.93	(2.38–6.51)	3.78	(2.01–7.09)	0.8910	2.28	(1.89–2.75)	1.92	(1.44–2.57)	2.52	(1.97–3.22)	0.162
Breastfeeding initiated within 1 h	1.76	(0.79–3.93)	1.80	(0.79–4.10)	1.71	(0.73–3.96)	0.7870	3.29	(2.60–4.18)	2.02	(1.41–2.91)	5.15	(3.74–7.10)	0.000
Only breastmilk given in first day								1.52	(1.22–1.91)	1.89	(1.35–2.65)	1.31	(0.97–1.78)	0.112
Exclusive breastfeeding in first 6 weeks after birth	0.80	(0.44–1.45)	0.70	(0.38–1.32)	1.32	(0.51–3.41)	0.1820	1.39	(1.13–1.72)	1.73	(1.25–2.37)	1.22	(0.92–1.62)	0.110





	Bangladesh							Malawi						
	Total		High SEP		Low SEP		Difference	Total		High SEP		Low SEP		Difference
	OR*	95% CI	OR*	95% CI	OR*	95% CI	*P*-value*	OR	(95% CI)	OR	(95% CI)	OR	(95% CI)	*P*-value*
Health care use														
3 + antenatal care visits to medical provider	1.55	(1.37–1.75)	1.60	(1.39–1.83)	1.39	(0.98–1.98)	0.476	0.94	(0.56–1.55)	0.92	(0.53–1.61)	0.87	(0.43–1.74)	0.843
Medical treatment sought for illness during pregnancy	1.18	(1.04–1.34)	1.22	(1.06–1.40)	0.98	(0.71–1.36)	0.235	–						
Institutional delivery	1.12	(0.97–1.28)	1.13	(0.97–1.31)	0.97	(0.62–1.53)	0.545	1.23	(0.69–2.19)	1.28	(0.67–2.42)	1.17	(0.55–2.52)	0.771
Institutional delivery for prolonged labour	–							–						
Home care: antenatal practices														
Iron intake	2.63	(2.32–2.98)	2.39	(2.08–2.75)	3.97	(2.96–5.32)	0.002	1.38	(0.47–4.08)	0.97	(0.32–2.98)	4.06	(0.94–17.54)	0.012
Home care: delivery practices														
Birth attendant washed hands	3.05	(2.39–3.87)	3.58	(2.68–4.79)	2.15	(1.38–3.37)	0.061	0.19	(0.06–0.53)	0.18	(0.07–0.47)	0.25	(0.08–0.73)	0.434
Clean delivery kit used	5.46	(4.51–6.62)	5.67	(4.59–7.01)	4.98	(3.13–7.90)	0.611	–						
Plastic sheet used	2.43	(2.09–2.83)	2.72	(2.29–3.23)	1.75	(1.28–2.41)	0.017	–						
Cord cut with new or boiled blade	2.46	(1.21–5.00)	2.95	(1.31–6.65)	1.40	(0.32–6.18)	0.390	–						
Cord tied with boiled thread	1.32	(1.13–1.53)	1.25	(1.05–1.49)	1.62	(1.16–2.24)	0.176	–						
Home care: postnatal practices														
Appropriate cord care (nothing applied on stump)	1.76	(1.48–2.09)	1.66	(1.37–2.01)	2.27	(1.57–3.28)	0.138	–						
Baby wrapped or put on the skin within 10 min	1.49	(1.28–1.73)	1.40	(1.18–1.66)	1.88	(1.36–2.62)	0.113	1.61	(0.39–6.71)	2.40	(0.49–11.80)	0.67	(0.11–4.31)	0.180
Baby placed on mother's skin within 30 min	0.93	(0.80–1.08)	0.85	(0.71–1.01)	1.26	(0.90–1.76)	0.038	–						
Baby not bathed in first 6 h after birth	1.74	(1.43–2.12)	1.76	(1.40–2.20)	1.61	(1.06–2.45)	0.711	1.51	(0.48–4.69)	1.76	(0.53–5.82)	0.72	(0.20–2.63)	0.013
Breastfeeding initiated within 1 h	2.55	(2.08–3.11)	2.59	(2.06–3.26)	2.45	(1.63–3.67)	0.812	1.79	(0.44–7.37)	2.32	(0.54–10.06)	1.49	(0.30–7.49)	0.340
Only breastmilk given in first day	2.28	(1.75–2.97)	2.42	(1.79–3.27)	1.79	(1.00–3.21)	0.370	–						
Exclusive breastfeeding in first 6 weeks after birth	1.29	(1.09–1.52)	1.30	(1.08–1.57)	1.26	(0.89–1.79)	0.888	–						


*P-value is for the difference in OR between lower and higher socioeconomic groups. Marginalization was used as indicator of socioeconomic position in the analyses for this table.

–, not available.

## Discussion

Our analysis shows that community-based women’s groups with high population coverage reduced neonatal mortality equitably and substantially across socioeconomic strata. The reductions were at least as strong among poor, illiterate and most marginalized groups as in better-off strata, with particularly pronounced effects on lower strata in the India trial. The intervention also improved home care practices among lower and higher strata, with no systematic differences between strata.

Problems measuring NMR are unlikely to explain our findings. Measuring NMR reliably in poor settings is challenging with surveys using retrospective birth histories, but in the four trials a combined population of over 1 million was prospectively followed up and outcomes were registered for every birth.

We used random effects logistic regression to account for clustering, following the main publication of two of the trials. Although this was less robust for trials with relatively few clusters and pair-matched trials compared with cluster-summary methods of analysis, we found it important to use the same analytical approach for all trials. Also, our models assume that the period effects are constant across clusters, which may be a strong assumption.

For the Malawi trial, we conducted a treatment group analysis comparing the ‘women’s group only’ arm with the ‘no intervention’ arm, resembling the other trials. Our findings should be seen in the light of discussions on analysis strategy.^25^ A factorial analysis of the Malawi trial showed no NMR effect, but this was possibly caused by baseline imbalances.^21^ We therefore decided to explore the ‘treatment group’ findings further.

The intervention effects observed among women who had not attended women’s groups could be due to selection effects. As primigravid women (whose infants are at a higher risk of neonatal death) were less likely to participate in the intervention,^13^ our estimates among non-attenders are perhaps an underestimation of the real effect. Nevertheless, potential selection effects make the effect estimates among non-attenders uncertain.

The trial areas were located in rural areas. Rural communities in developing countries are not, however, homogeneously poor; there is still important socioeconomic differentiation by literacy, wealth and caste. These differences influence uptake of interventions, health care and healthy behaviours; in poor countries, inequalities of uptake are often very large.^26^

Our findings are important as, based on the inverse equity hypothesis^27^ and Tudor Hart’s inverse care law,^28^ we expect interventions – even ‘simple’ behavioural interventions – to reach higher socioeconomic groups better and to only benefit lower strata once higher strata reach a plateau beyond which further improvements are difficult to achieve. The reasons for this include inequitable intervention availability, elite capture at the community level when services and interventions are made available and a slower diffusion of behaviour change among lower socioeconomic strata.^7^^,^^29^^,^^30^ This is also reflected in, for example, substantial inequalities in uptake of low-cost interventions such as oral rehydration therapy for children with diarrhoea, with lower uptake among lower socioeconomic groups in many low- and middle-income countries.^26^ There are indications that community-based interventions can reinforce existing social hierarchies.^8–11^^,^^31^ Conversely, our findings show that lower and higher socioeconomic strata can experience survival and behavioural improvements in tandem.

The equitable distribution of the women’s group effects is important because little is known about how to reduce health inequalities.^32^^,^^33^ Health inequalities are a persistent problem in poor and rich countries, and few interventions are effective in reducing them. There is some evidence that home visits by community health workers can improve equity in antenatal and postnatal practices and coverage of childhood vaccinations,^34^^,^^35^ but studies examining differential impacts on neonatal mortality across socioeconomic groups remain extremely rare.^32^^,^^33^^,^^36^

An important contributor to the equitable intervention effects is probably the equitable reach of the intervention across strata, on which we reported in a separate paper.^13^ In that paper, we describe how equitable attendance at the women’s groups is related to intervention design, which included a combination of universal coverage (the groups were open to everyone who shared a concern about maternal and newborn health) and ‘soft targeting’ — meeting in places and at times that are convenient to lower socioeconomic strata, using methods and language that engage women with low levels of literacy and that involve the entire community and addressing issues around pregnancy and delivery that are important to everyone.^2^^,^^13^ And importantly, the women’s groups’ strategies focused on simple behavioural changes in the home, which were accessible and affordable to poor and illiterate families, and which could lead to survival benefits especially in contexts where home deliveries are common. In our current paper, we show that survival improvements were also observed among newborn infants of women who had not attended groups, in lower and higher strata, suggesting a diffusion of the group messages and actions across socioeconomic layers, not just the better-off.

The intervention effect across social strata was also observed for early NMR, which is important as early NMR is resistant to decline.^37^ The effect on early NMR is notable given that improvements in the institutional delivery rate were limited or absent. This suggests that behavioural improvements around delivery in the home, including prevention of infection and hypothermia, and handling of premature and perhaps even asphyxiated infants, played a role. Strong effects on late NMR were only observed for lower strata in the last trial year, suggesting that infection prevention and improvement in feeding practices were perhaps especially important among lower strata. So although the intervention design contributed to equitable uptake, its particularly strong mortality impacts among lower strata were possibly facilitated by socioeconomic differences in cause-of-death distribution, with arguably a greater importance of infections as cause of death among the poor.^38^

### Policy implications

Women’s groups reduced newborn mortality among all socioeconomic layers within rural populations, especially among the most marginalized. The intervention can be scaled up through community health workers as group facilitators. Where a country-wide network of such health workers exits, such as the Accredited Social Health Activists (ASHAs) in India, the potential for scaling up is large. Currently, a trial to test the impact of women’s groups facilitated by ASHAs is ongoing.^39^ The potential contribution of women’s groups to a world without preventable newborn deaths, as envisaged in the Every Newborn Action Plan, can be large if our conclusions also hold in a programmatic (rather than trial) setting.^1^

We recommend that the women’s group intervention is implemented together with health system interventions to improve equitable uptake of professional maternity care, such as conditional cash transfers, voucher schemes and other measures to improve equitable uptake and quality of maternity care.^40–42^

The women’s group intervention is only effective when implemented well, i.e. with sufficient coverage, duration and intensity, as demonstrated in three other trials.^15^^,^^16^ Achieving high coverage is partly related to resources and knowledge about the importance of coverage, and partly related to context. Achieving high coverage in urban slum areas, with high migration levels and little space to meet, is difficult, as became evident in the Mumbai trial.^16^ In rural areas with high mortality levels, women’s groups, when implemented well, seem a safe bet to reduce newborn mortality among the poor and the less poor.

The principles of the women’s group intervention are arguably amenable to wider implementation. Principles for ensuring equitable intervention effects include universal coverage – making the intervention open to everyone – combined with soft-targeting to ensure uptake among lower strata, involving the wider community and thus ensuring diffusion, while focusing on those practices or problems that are important contributors to mortality and while also addressing the wider determinants of health.^2^

### Conclusion

Community-based women’s groups with high population coverage benefit the survival chances of all newborn infants, and perhaps especially those born into the most deprived households.

## Funding

This work was primarily supported by the Economic and Social Research Council and the Department for International Development (grant number ES/I033572/1). Additional support was provided by a Wellcome Trust Strategic Award (award number: 085417MA/Z/08/Z). T.A.J.H. was also supported by an EUR Research Excellence Initiative grant. The funders had no role in study design, data collection and analysis, decision to publish, or preparation of the manuscript.

## Supplementary Material

Supplementary Data
