# Supplementary material for: The equity impact of community women’s groups to reduce neonatal mortality: a meta-analysis of four cluster randomized trials
Source: Int J Epidemiol. 2017 Aug 25;48(1):168–82. doi: 10.1093/ije/dyx160 (PMC6380297; doi:10.1093/ije/dyx160)
Supplement: Supplementary Data [file dyx160_supp.zip › dyx160-suppl_data/dyx160_Supplementary_Table_2.docx]

**Table S2: Intervention effects on the neonatal mortality rate for lower and higher socio-economic groups, per trial and pooled estimates, for the last study year, all six trials**

|  | **Pooled estimates** | | | **Nepal** | | | **India (rural)** | | | **India (urban)** | | | **Bangladesh (1)** | | | **Bangladesh** | | | **Malawi** | | |
| --- | --- | --- | --- | --- | --- | --- | --- | --- | --- | --- | --- | --- | --- | --- | --- | --- | --- | --- | --- | --- | --- |
|  | OR* | 95%CI | p-value** | OR* | 95%CI | p-value** | OR* | 95%CI | p-value** |  |  |  |  |  |  | OR* | 95%CI | p-value** | OR* | 95%CI | p-value** |
| **Total** | 0.67 | (0.49;0.91) | 0.010 | 0.64 | (0.40;1.03) | 0.064 | 0.46 | (0.32;0.65) | 0.000 | 1.09 | (0.46;2.60) | 0.841 | 1.02 | (0.83;1.26) | 0.830 | 0.45 | (0.31;0.64) | 0.000 | 0.80 | (0.43;1.51) | 0.498 |
|  |  |  |  |  |  |  |  |  |  |  |  |  |  |  |  |  |  |  |  |  |  |
| **Marginalisation** |  |  |  |  |  |  |  |  |  |  |  |  |  |  |  |  |  |  |  |  |  |
| less marginalised | 0.84 | (0.64;1.10) | 0.056 | 0.82 | (0.45;1.50) | 0.240 | 0.82 | (0.48;1.41) | 0.010 | 1.21 | (0.46;3.17) | 0.752 | 1.15 | (0.90;1.46) | 0.055 | 0.49 | (0.32;0.73) | 0.413 | 0.85 | (0.43;1.70) | 0.652 |
| most marginalised | 0.48 | (0.30;0.79) |  | 0.45 | (0.20;1.01) |  | 0.32 | (0.20;0.51) |  | 0.95 | (0.26;3.46) |  | 0.72 | (0.47;1.09) |  | 0.33 | (0.14;0.76) |  | 0.67 | (0.25;1.79) |  |
|  |  |  |  |  |  |  |  |  |  |  |  |  |  |  |  |  |  |  |  |  |  |
| **Literacy** |  |  |  |  |  |  |  |  |  |  |  |  |  |  |  |  |  |  |  |  |  |
| literate | 0.79 | (0.57;1.10) | 0.435 | 0.50 | (0.20;1.27) | 0.551 | 0.71 | (0.33;1.50) | 0.240 | 1.59 | (0.55;4.63) | 0.334 | 1.15 | (0.88;1.49) | 0.161 | 0.46 | (0.30;0.70) | 0.798 | 0.91 | (0.43;1.94) | 0.547 |
| illiterate | 0.61 | (0.35;1.07) |  | 0.70 | (0.40;1.23) |  | 0.42 | (0.28;0.63) |  | 0.78 | (0.25;2.50) |  | 0.84 | (0.59;1.19) |  | 0.41 | (0.20;0.84) |  | 0.66 | (0.27;1.59) |  |
|  |  |  |  |  |  |  |  |  |  |  |  |  |  |  |  |  |  |  |  |  |  |
| **Economic status** |  |  |  |  |  |  |  |  |  |  |  |  |  |  |  |  |  |  |  |  |  |
| less poor | 0.75 | (0.53;1.08) | 0.656 | 0.91 | (0.47;1.75) | 0.143 | 0.76 | (0.41;1.42) | 0.055 | 1.01 | (0.33;3.11) | 0.848 | 1.12 | (0.83;1.51) | 0.307 | 0.31 | (0.17;0.57) | 0.120 | 0.75 | (0.30;1.88) | 0.864 |
| poorest | 0.64 | (0.35;1.18) |  | 0.44 | (0.22;0.90) |  | 0.36 | (0.24;0.56) |  | 1.16 | (0.39;3.46) |  | 0.90 | (0.68;1.21) |  | 0.57 | (0.36;0.90) |  | 0.83 | (0.39;1.76) |  |

India (urban): 24+24 clusters, intervention population: 283000. 2% of women who delivered a baby during the trial period attended the women’s groups.

Bangladesh (1): 9+9 clusters, intervention population: 500000. 3% of women who delivered a baby during the trial period attended the women’s groups.

*Note on measurement of economic status:* Principal component analysis based asset index. Included assets were as follows: *India (urban)*: electricity telephone fan tv fridge radio bicycle, *Bangladesh (1)*: electricity generator telephone fan tv fridge radio bicycle.
