# Supplementary material for: The equity impact of community women’s groups to reduce neonatal mortality: a meta-analysis of four cluster randomized trials
Source: Int J Epidemiol. 2017 Aug 25;48(1):168–82. doi: 10.1093/ije/dyx160 (PMC6380297; doi:10.1093/ije/dyx160)
Supplement: Supplementary Data [file dyx160_supp.zip › dyx160-suppl_data/dyx160_Supplementary_Table_4.docx]

**Table S4: Intervention effects on the neonatal mortality rate for lower and higher socio-economic groups, per trial and pooled estimates, for the last study year, among infants of women that had not attended women’s groups**

|  | **Pooled estimates** | | | **Nepal** | | | **India** | | | **Bangladesh** | | | **Malawi** | | |
| --- | --- | --- | --- | --- | --- | --- | --- | --- | --- | --- | --- | --- | --- | --- | --- |
|  | OR* | 95%CI | p-value** | OR* | 95%CI | p-value** | OR* | 95%CI | p-value** | OR* | 95%CI | p-value** | OR* | 95%CI | p-value** |
| **Total** | 0.55 | (0.40;0.76) | 0.000 | 0.82 | (0.49;1.36) | 0.441 | 0.47 | (0.31;0.70) | 0.000 | 0.40 | (0.26;0.61) | 0.000 | 0.85 | (0.39;1.85) | 0.678 |
|  |  |  |  |  |  |  |  |  |  |  |  |  |  |  |  |
| **Marginalisation** |  |  |  |  |  |  |  |  |  |  |  |  |  |  |  |
| less marginalised | 0.69 | (0.48;0.98) | 0.069 | 1.05 | (0.55;2.01) | 0.281 | 0.90 | (0.49;1.66) | 0.010 | 0.44 | (0.28;0.71) | 0.390 | 0.75 | (0.31;1.82) | 0.494 |
| most marginalised | 0.39 | (0.24;0.64) |  | 0.58 | (0.25;1.37) |  | 0.30 | (0.17;0.53) |  | 0.27 | (0.10;0.75) |  | 1.17 | (0.37;3.66) |  |
|  |  |  |  |  |  |  |  |  |  |  |  |  |  |  |  |
| **Literacy** |  |  |  |  |  |  |  |  |  |  |  |  |  |  |  |
| literate | 0.54 | (0.38;0.77) | 0.917 | 0.73 | (0.28;1.92) | 0.809 | 0.91 | (0.40;2.04) | 0.079 | 0.40 | (0.25;0.66) | 0.994 | 0.73 | (0.27;1.94) | 0.543 |
| illiterate | 0.53 | (0.38;0.73) |  | 0.84 | (0.46;1.55) |  | 0.39 | (0.24;0.63) |  | 0.40 | (0.17;0.93) |  | 1.06 | (0.39;2.93) |  |
|  |  |  |  |  |  |  |  |  |  |  |  |  |  |  |  |
| **Economic status** |  |  |  |  |  |  |  |  |  |  |  |  |  |  |  |
| less poor | 0.65 | (0.45;0.93) | 0.211 | 1.12 | (0.55;2.29) | 0.241 | 0.75 | (0.37;1.53) | 0.122 | 0.34 | (0.18;0.67) | 0.518 | 0.91 | (0.30;2.80) | 0.973 |
| poorest | 0.48 | (0.35;0.65) |  | 0.61 | (0.29;1.27) |  | 0.38 | (0.23;0.62) |  | 0.46 | (0.26;0.79) |  | 0.89 | (0.35;2.27) |  |

* The ratio of the odds of neonatal mortality in the intervention compared to the control areas adjusted for baseline differences in neonatal mortality. For the Nepal trial, it was not possible to adjust for baseline mortality differences.

** P-value for the test on difference in OR between lowest and highest socio-economic groups. For the total population, it gives the p-value for the difference between intervention and control.
