# Supplementary material for: The equity impact of community women’s groups to reduce neonatal mortality: a meta-analysis of four cluster randomized trials
Source: Int J Epidemiol. 2017 Aug 25;48(1):168–82. doi: 10.1093/ije/dyx160 (PMC6380297; doi:10.1093/ije/dyx160)
Supplement: Supplementary Data [file dyx160_supp.zip › dyx160-suppl_data/dyx160_Supplementary_Table_3.docx]

**Table S3: Intervention effects on the neonatal mortality rate for lower and higher socio-economic groups, per trial and pooled estimates, for the last two study years**

|  | **Pooled estimates** | | | **Nepal** | | | **India** | | | **Bangladesh** | | | **Malawi** | | |
| --- | --- | --- | --- | --- | --- | --- | --- | --- | --- | --- | --- | --- | --- | --- | --- |
|  | OR* | 95%CI | p-value** | OR* | 95%CI | p-value** | OR* | 95%CI | p-value** | OR* | 95%CI | p-value** | OR* | 95%CI | p-value** |
| **Total** | 0.60 | (0.52;0.71) | 0.000 | 0.71 | (0.53;0.96) | 0.026 | 0.49 | (0.36;0.66) | 0.000 | 0.60 | (0.45;0.80) | 0.001 | 0.62 | (0.40;0.96) | 0.034 |
|  |  |  |  |  |  |  |  |  |  |  |  |  |  |  |  |
| **Marginalisation** |  |  |  |  |  |  |  |  |  |  |  |  |  |  |  |
| less marginalised | 0.68 | (0.55;0.83) | 0.116 | 0.78 | (0.52;1.16) | 0.832 | 0.74 | (0.45;1.21) | 0.051 | 0.62 | (0.44;0.86) | 0.632 | 0.62 | (0.39;1.00) | 0.962 |
| most marginalised | 0.52 | (0.41;0.67) |  | 0.73 | (0.46;1.14) |  | 0.40 | (0.27;0.59) |  | 0.52 | (0.28;0.97) |  | 0.61 | (0.30;1.27) |  |
|  |  |  |  |  |  |  |  |  |  |  |  |  |  |  |  |
| **Literacy** |  |  |  |  |  |  |  |  |  |  |  |  |  |  |  |
| literate | 0.61 | (0.48;0.78) | 0.923 | 0.50 | (0.27;0.92) | 0.134 | 0.77 | (0.39;1.53) | 0.157 | 0.60 | (0.42;0.85) | 0.959 | 0.65 | (0.39;1.09) | 0.753 |
| illiterate | 0.60 | (0.49;0.74) |  | 0.85 | (0.60;1.21) |  | 0.45 | (0.32;0.63) |  | 0.59 | (0.34;1.00) |  | 0.58 | (0.31;1.09) |  |
|  |  |  |  |  |  |  |  |  |  |  |  |  |  |  |  |
| **Economic status** |  |  |  |  |  |  |  |  |  |  |  |  |  |  |  |
| less poor | 0.64 | (0.49;0.82) | 0.549 | 0.81 | (0.52;1.27) | 0.479 | 0.60 | (0.34;1.05) | 0.408 | 0.48 | (0.30;0.77) | 0.236 | 0.72 | (0.39;1.33) | 0.479 |
| poorest | 0.58 | (0.47;0.70) |  | 0.65 | (0.44;0.97) |  | 0.45 | (0.31;0.65) |  | 0.69 | (0.48;1.00) |  | 0.55 | (0.33;0.92) |  |

* The ratio of the odds of neonatal mortality in the intervention compared to the control areas adjusted for baseline differences.

** P-value for the test on difference in OR between lowest and highest socio-economic groups. For the total population, it gives the p-value for the difference between intervention and control.

P-values for heterogeneity test in pooled analysis: marginalisation (p=0.3985), literacy (p=0.1737), economic status (p=0.3235).
