# Supplementary material for: The equity impact of community women’s groups to reduce neonatal mortality: a meta-analysis of four cluster randomized trials
Source: Int J Epidemiol. 2017 Aug 25;48(1):168–82. doi: 10.1093/ije/dyx160 (PMC6380297; doi:10.1093/ije/dyx160)
Supplement: Supplementary Data [file dyx160_supp.zip › dyx160-suppl_data/dyx160_Supplementary_Table_1.docx]

**Table S1: Overview of six cluster randomised trials with participatory women’s groups**

| **Country** (implementing organisation) | **Location** | **# clusters** intervention + control  (total study population) | **RCT design** | **# women’s groups**  (% women attending the groups of those who delivered a baby, by study year) | **Trial period**  Baseline (B) and intervention period (by intervention year (Y)) | **Interview response rate** in intervention (I) & control areas (C) |
| --- | --- | --- | --- | --- | --- | --- |
| Nepal  (MIRA-Makwanpur) | Makwanpur district | 12 +12  (169,776) | Matched.  Closed cohort of women aged 15-49 years old. | 111  (around 38%) | B: no prospective baseline  Y1: 1 Nov. 2001 - 31 Oct. 2002  Y2: 1 Nov. 2002 - 31 Oct. 2003 | I: 95%  C: 95% |
| India  (Ekjut) | 3 districts in the states of Odisha and Jharkhand | 18+18  (228,000) | Stratified.  Open cohort of women aged 15-49 years old. | 244  (increasing over the years from 14% to 54%) | B: 21 Nov. 2004 – 30 July 2005  Y1: 31 July 2005 – 30 July 2006  Y2: 31 July 2006 – 30 July 2007  Y3: 31 July 2007 – 30 July 2008 | I: > 99%  C: 98% |
| India (SNEHA) | Mumbai | 24+24  (283,000) | Blocked (8 per each of 6 municipal wards)  Open cohort of women aged 15-49 years old. | 244 (2%) | B: 1 Oct 2005 – 30 Sep 2006  Y1: 1 Oct 2006 – 30 Sep 2007  Y2: 1 Oct 2007 – 30 Sep 2008  Y3: 1 Oct 2008 – 30 Sep 2009 | I: 84%  C: 83% |
| Bangladesh  (PCP-BADAS) Trial 1 | Bogra, Faridpur, Moulvibazar district | 9+9  (500,000) | Stratified.  Open cohort of women aged 15-49 years old. | 162 (3%) | B: no prospective baseline  Y1: 1 Feb 2005 – 31 Dec 2005  Y2: 1 Jan 2006 – 31 Dec 2006  Y3: 1 Jan 2007 – 31 Dec 2007 | I: 84%  C: 82% |
| Bangladesh  (PCP-BADAS)  Trial 2 | Bogra, Faridpur, Moulvibazar district | 9+9  (500,000) | Stratified.  Open cohort of women aged 15-49 years old. | 810  (increasing over the years from 31% to 36%) | B: 1 Jan. 2008 – 31 Dec. 2008  Y1: 1 July 2009 – 30 June 2010  Y2: 1 July 2010 – 30 June 2011 | I: 99%  C: 99% |
| Malawi  (MaiMwana) | Mchinji district | 12+12  (183,000) | Factorial; stratified.  Open cohort of women aged 15-49 years old. | 207  (increasing over the years from 53% to 57%) | B: 1 Jan. 2005 – 31 Jan 2006 (*)  Y1: 1 Feb. 2006 – 31 Jan 2007  Y2: 1 Feb. 2007 – 31 Jan 2008  Y3: 1 Feb. 2008 – 31 Jan 2009 | I: 99%  C: 99% |

(*) The MaiMwana trial paper used a baseline until 30 June 2005 and used 1 July 2005 – 31 Jan 2006 as ‘run-in’ period, which was excluded from the trial analysis. There were concerns that the baseline period was too short to adequately measure baseline mortality. We therefore combined the ‘run-in period’, in which not much intervention activities were implemented yet, with the baseline period in our analyses.
